# Supplementary material for: Experimental investigation into the effect of compliance of a mock aorta on cardiac performance
Source: PLoS One. 2020 Oct 12;15(10):e0239604. doi: 10.1371/journal.pone.0239604 (PMC7549783; doi:10.1371/journal.pone.0239604)
Supplement: Attachment — Submitted filename: Authors_Rebuttal_PLOS1_final.docx [file pone.0239604.s001.docx]

# REPLY TO REVIEWER COMMENTS

Report on “Experimental investigation into the effect of compliance of a mock aorta on cardiac performance ".

Note: Reviewer’s comments have been copied from the email sent by the editor.

# Reply to comments from Reviewer #1

*Reviewer #1:*

This paper investigates the effects of a rigid tube and a compliant mock aorta placed in the left side of an ex-vivo heart perfusion (EVHP) on pressure waveforms, centerline velocity, tube distension, and energy demand on the pump. It concludes that given the healthier physiologic pressure waveforms, steadier downstream flow, and reduced energetic demands on the pump for the compliant mock aorta, it provides the experimental verifications of Windkessel theory and suggests the use of mock aorta in the EVHP system.

I believe this is an interesting study since it provides experimental evidence on the effects of elastic aorta model compared to a rigid one. The value of this paper from fluid mechanics perspective justifies its publication after some minor issues are addressed:

**Authors’ Reply**

Thank you for reviewing the paper and highlighting the strengths of the manuscript.

1. The discussion on the cardiac performance can be extended to other parameters rather than pump energy output, in order to better evaluate the healthier physiological condition. Parameters such as ejection fraction or h-efficiency (Song & Borazjani, 2015) can be helpful.

**Authors’ Reply**

While there are differences between the two works, we agree that the discussion about efficiency adds value to the paper. We therefore highlighted the distinction between the work in our paper and an indicator related to physiological conditions. The focus of our research is to understand the pumping energy itself that is required in optimization of EVHP system. Metrics such as ejection fraction are very much a reflection of the intrinsic properties of the left ventricle itself (i.e. if ejection fraction is < 50%, left ventricular muscle is weak and unable to pump effectively regardless of the environment – this is heart failure). We were interested in the impact of flow loop conditions on the pump/heart without looking at physiological differences between organs themselves or any sort of pathophysiology. Therefore, we discussed the suggested reference in the introduction section of the revised paper and explained our choice which is related EVHP performance.

**Change in the paper:**

While there is research in the literature that uses metrics such as ejection fraction to assess the physiological conditions of the heart (22), there is limited research into how the circulatory conditions affect heart performance in an ex-vivo setup.

2. It does not seem necessary to talk about the Womersley number to provide the reasoning for using the unsteady Bernoulli equations; however, the authors did not provide the Womersley number related to their own experiments to justify the use of unsteady Bernoulli equation. In addition, the first sentence of second paragraph of page 6 is too general and is not a fact. Also, the reference number 28 suggested a limit value of α=1 not 10.

**Authors’ Reply**

The limit value was corrected in the text. Regarding the Womersley number, as discussed in the manuscript, cycle time varied depending on the afterload conditions of the system which results in different Womersley numbers. In the experiments changing the pulse frequency from 1 to 2.33 Hz resulted in α values of 16 to 24. This has been added to the revised manuscript as per the recommendation.

**Change in the manuscript**

As a result of the VAD driving pressure being fixed in these experiments, the pulse frequency and therefore cycle time varied depending on the afterload conditions of the system which were primarily regulated by back pressure generated by the afterload pump. Changing the pulse frequency from 1 to 2.33 Hz results in Womersley numbers that vary from 16 to 24.

3. It seems that friction losses should be included in Eq. 2 as it is included in Eq. 3. Since the authors choose to start from general form (Eq. 2), it would be better to provide all the simplification and the underlying reasons for them. For example, it might not be clear for the readers how the fourth term in Eq. 2 is neglected.

**Authors’ Reply**

The friction losses were added to Eq. 2 and the details discussed as recommended.

$p_{1}+\rho gz_{1}+\frac{\rho{u_{1}}^{2}}{2}+\int_{0}^{1} \frac{du}{dt}ds=p_{2}+\rho gz_{2}+\frac{\rho{u_{2}}^{2}}{2}+\int_{0}^{2} \frac{du}{dt}ds+p_{losses}$

Neglecting the variation of the hydrostatic pressure, Eq. 2 can be written as follows:

$\Delta p\left( t \right)-p_{\text{losses}}=\int_{1}^{2} \frac{du}{dt}ds+\frac{1}{2}\rho\left( {u_{2}}^{2}-{u_{1}}^{2} \right)$

With the assumption that the acceleration varies with area in the same way as $u$ (Pasipoularides et al. (1984)), the following equation is obtained:

$\Delta p\left( t \right)-p_{\text{losses}}=\rho\text{L}\frac{du_{2}}{dt}+\frac{1}{2}\rho\left( {u_{2}}^{2}-{u_{1}}^{2} \right)$

**Changes in the paper**

A form of the energy equation for incompressible flow was used to …

**Changes in the paper**

$$p_{1}+\rho gz_{1}+\frac{\rho{u_{1}}^{2}}{2}+\int_{0}^{1} \frac{du}{dt}ds=p_{2}+\rho gz_{2}+\frac{\rho{u_{2}}^{2}}{2}+\int_{0}^{2} \frac{du}{dt}ds+p_{losses}$$

(2)

$p_{\text{losses}}$ are the frictional losses that occur along the tube length (Pa), and $\text{L}$ is the length of the fluid volume being accelerated (m).

Using the assumptions that the height change between the points has a negligible effect on the flow and that the tube has a uniform diameter between points 1 and 2 and using the assumption that the acceleration varies with area in the same way as $u$ (32), the pressure differential driving the flow is described as:

$$\Delta p\left( t \right)-p_{\text{losses}}=\int_{1}^{2} \frac{du}{dt}ds+\frac{1}{2}\rho\left( {u_{2}}^{2}-{u_{1}}^{2} \right)$$

(3)

$$\Delta p\left( t \right)-p_{\text{losses}}=\rho\text{L}\frac{du_{2}}{dt}+\frac{1}{2}\rho\left( {u_{2}}^{2}-{u_{1}}^{2} \right)$$

(4)

4. The structural properties of the material employed to make the mock aorta is not addressed. Please provide the material properties in the manuscript or refer to appropriate literature.

**Authors’ Reply**

The properties have been added to the manuscript and the proper reference was provided.

**Change in the manuscript**

The silicone has a specific gravity of 1.07 g/cc and an elastic modulus of 12 psi (54,55).

5. It might be helpful to better present the experimental setup by clarifying the differences between the current experimental setup (Fig 4) and EVHP system (Fig 2) at the beginning of the experimental setup section.

**Authors’ Reply**

A discussion has been added to the manuscript to for comparison between Fig 2 and Fig 4.

**Change in the manuscript**

The experimental setup mimics the left flow loop shown in Fig 2 (the flow path between the left ventricle and the reservoir). The notable differences between the experimental setup and the EVHP system shown in Fig 2 are the utilization of separate preload and afterload reservoirs, using the VAD instead of a heart and the absence of a preload pump for the VAD. The preload pump has not been used in the experimental setup since there is no need to sustain constant left atrial pressure such as what is needed in the EVHP system.

6. For the averaging of the centerline velocity, to be clearer, it is better to first present the underlying reason and then providing the averaging methodology.

**Authors’ Reply**

The related paragraph was revised to address this comment.

**Change in the manuscript**

Vector values were averaged over a region to prevent spatial variations from having a significant impact on the centerline velocity calculations. Averaging centreline velocities was undertaken over a region that spanned the full axial length of the image and a region either side of the centreline for *r/D* = 0.413 to 0.588. There was often the presence of secondary flows as shown in Fig 5 and therefore non-uniformities in the axial direction occurred during diastole.

7. It is recommended to add a new section for pump energy calculations rather than having it under the experimental setup section.

**Authors’ Reply**

A new section was used in the revised manuscript to describe calculation of pump energy.

**Change in the manuscript**

Subsection title: Pumping Performance

8. In Fig. 6, it is not clear how the negative values of diastolic pressure for rigid tube is measured; however, in the manuscript, the diastolic pressure is reported 68.1 for this case, which does not consider the negative parts of the pressure waveform and is inconsistent with the definition of diastolic pressure presented on page 6.

**Authors’ Reply**

The negative values in Fig 6 can be expressed as a reflection of the wave that impeding the VAD outlet valve at time it is supposed to be closed. This pressure is measured using the pressure sensors that are calibrated to provide a range pressures from negative to positive values. The diastolic pressure in Fig 6 for rigid tube therefore is the point that that the pressure wave starts to form the next systolic wave. The pressure at this point is 68.1 mmHg as described in the manuscript. To avoid further confusion the figure is revised with symbols on it to explain the critical points.

**Changes in the manuscript:** Figure 6 has been revised as below.

Figure caption: $P_{s}$ and $P_{d}$ locations are shown for rigid and compliant cases with the same colour codes.

9. In Fig. 7, horizontal axes must be shifted 0.5 of the normalized time to fix its zero value to the beginning of the systole. In addition, the sharp momentary increase after relaxation is not clear in the plot. Also, please add the appropriate legends to Fig.7 to distinguish the contributors to the tube expansion.

**Authors’ Reply**

The issue has been fixed in the revised manuscript. The description of the contributors can be found in the figure caption.

**Change in the manuscript:** New figure

10. The description of the indexing cycles for Fig. 8 is not clear. Does the zero time instant set to be consistent with the pressure plots, as they are plotted together at later figures. Please clarify this sentence: “Both response cycles are indexed to begin at the image corresponding to the first diastolic minimum after the Camera 2 trigger signal.”

**Authors’ Reply**

The statement is now further explained in the paper to clarify the discussion.

**Change in the manuscript**

The zero time instant defined in the Fig 8 corresponds to the zero time of the pressure signal shown in Fig 6.

11. In fig. 9, Normalized pressure does not show negative value; however its absolute values does (Fig.6). In addition, pressure values at the beginning and end of the cycle do not match. Please clarify/fix it.

**Authors’ Reply**

The pressure data has been updated and the plot was properly corrected, so it matches Fig. 6.

12. The dicrotic notch for pressure plot of Fig 10 is reported as 0.45, which is not consistent with the previous value of 0.4 on page 16 and the definition of dicrotic notch presented on page 6.

**Authors’ Reply**

The value provided earlier on page 16 was an approximation that is described later in the manuscript using Fig 10. This, however, is changed in both places in the manuscript to prevent readers from any confusion. According to Fig 6, dicrotic notch is considered as a secondary upstroke in the descending part of the pressure wave and this definition has been used to best approximate the dicrotic notch in the experiments.

13. It is better to use the absolute value of the peak velocity ratio in Fig. 11, in order to visually show the increasing influence of negative centerline velocity, i.e., reverse flow.

**Authors’ Reply**

The absolute value of the peak velocity ratio is now used in Fig. 11 (and also Fig. 12) in order to reflect the increasing influence of the negative centerline velocity with the rigid tube as compared to the decreasing influence of the negative centerline velocity with the compliant tube.

**Change in the manuscript**

Figure 11 and caption have been revised.

In the text: On the same figure, the absolute values of the ratio between the peak reverse flow during the cycle to the peak positive flow $\left| \text{v}_{\text{pk}\text{,(-)}}\text{/}\text{v}_{\text{pk}\text{,(+)}} \right|$is presented. The absolute ratio reflects the increasing influence of the negative centerline velocity with the rigid tube as compared to the decreasing influence of the negative centerline velocity with the compliant tube.

Fig 11: Plot representing the peak positive downstream centerline velocity, peak negative centerline velocity and absolute peak velocities ratio (peak negative to peak positive) as function of the afterload pump speed for the rigid case

Fig 12: Plot representing the peak positive downstream centerline velocity, peak negative centerline velocity and absolute peak velocities ratio (peak negative to peak positive) as function of mock aorta distensibility for the compliant case where $\text{P}_{\text{ch}}\text{ = 103 mmHg}$.

14. Why there is a jump in data provided for Fig 12? Is there any experimental difficulty in gathering the data for this interval?

**Authors’ Reply**

The jump in the values are related to the nature of the experiments that shows the non-linear relation between afterload pump speed and the distension in the tube. The experimental difficulty that stops performing further cases is the limit in the chamber pressure size (to allow further distention) and the safety concerns over increasing the pressure inside the chamber pressure.

Some other comments:

1. Please revise the last sentence of the second paragraph on page 4. It seems that both impacts are basically the same and it strike me as a repetition: the impact of the lack of the elastic response and the impact of adding a compliant aorta.

**Authors’ Reply**

This comment has been addressed and the statement expressed not to be read as a repetition.

2. There are minor typos and inconsistencies in references such as reference number 27.

**Authors’ Reply**

The issue has been addressed. The entire paper was reviewed for any typos, as well.

3. The cross-reference to table 1 on page 21 needs to be corrected.

**Authors’ Reply**

This comment has been addressed.

# Reply to comments from Reviewer #2

*Reviewer #2:*

The study addresses an important problem. While the authors have done a good job with the study and composed the manuscript well, the current state of the manuscript needs significant improvement. The manuscript could use some level of reorganization, rewriting so that it is clearer to the reader. Several data acquisition campaigns have been undertaken, but the resulting data analysis could be improved, the analysis of the high-fidelity data acquired (for example the PIV) could be analyzed in a more in-depth manner.

**Authors’ Reply**

Thank you for reviewing the paper and for highlighting the strengths of the research work. The reviewer’s feedback is appreciated.

The following are some specific comments:

Please present the flow rate curve along with the pressure curve. Getting the right cardiac output is important.

**Authors’ Reply**

The flow rate curve has been added to the manuscript and the cardiac output has been calculated based on the flow rate curve and the systolic time. The details has been discussed in the paper as below.

**Change in the manuscript**

Fig 10(b) shows the flow rate values, calculated from the velocity data, along with the pressure data for the compliant case. By integrating the flow rate curve over systolic ejection time, a stroke volume of 60.5 mL was obtained. Using the pulse frequency of 67.2 bpm (1.12 Hz), cardiac output was calculated to be 4.1 L/min.

Fig. 10’s caption updated accordingly.

How about PIV data during systole? What is the centerline velocity during systole?

**Authors’ Reply**

To address the comment, a velocity field during a systole phase was also added to the revised manuscript as Fig. 5(b). The centerline velocity distribution is shown during both systole and diastole (Fig 8-10). These have been extracted from PIV results.

**Change in the manuscript**

A velocity field during a systole phase was also added and the figure caption has been edited.

Fig 5: (a) Vector field images with a background color map of streamwise velocity component obtained from processing Camera 2 images during diastole, (b) velocity filed during a systole phase.

PIV uncertainty estimation has been conducted, but only limited number of factors are considered. See Raffel et al (2013) or Raghav et al (2018) for more details.

**Authors’ Reply**

Further PIV uncertainty analysis of the PIV results is presented in the revised manuscript following the recommendation.

**Changes in the manuscript**

Based on the evaluation of the global bias error and the statistical uncertainty related to the data scattering around the mean values, the uncertainty of the PIV measurement is estimated to be in the range of ±0.2% to ±0.8% for the velocity components, and in the range of ±1% to ±4% for the corresponding rms velocity (64–66).

Fig 5 please mention exact time point during diastole? The positive velocity near the wall region is unrealistic, if r/D =0 and 1 are the walls

**Authors’ Reply**

The exact time for the systole and diastole has been shown in the revised manuscript on Fig. 6. The velocity field shown in Fig. 5 corresponds to the end of the diastole; this can explain the positive velocity near the wall for a short time window. This can be more explained by looking at Fig. 6. The negative values in Fig 6 can be expressed as a reflection of the wave that impeding the VAD outlet valve at time it is supposed to be closed. The diastolic pressure in Fig. 6 therefore is the point that that the pressure wave starts to form the next systolic wave.

**Changes in the manuscript:** Fig. 6 has been revised as below.

Figure caption: $P_{s}$ and $P_{d}$ locations are shown for rigid and compliant cases with the same colour codes.

With regards to the unsteady Bernoulli formulation, please discuss how it compares to Donati et al Circulation (2017).

**Authors’ Reply**

The work of the suggested reference (Donati et al, Circ Cardiovasc Imaging, 2017) which measures the velocity for ascending aorta is very similar to our work since it accounts for unsteadiness, advection and friction losses. Donati et al. are criticizing the approximation usually done to calculate pressure drop by neglecting the unsteadiness and the friction losses, which is also what we are doing. Donati et al. highlight that additional contributions to the flow due to the valve dis-functioning can change the flow behaviour and the pressure drop. This is different from our work that is focused on the resulting flow downstream from the mock aorta and visualize the flow inside the mock aorta. Therefore, to address the comment, Donati et al. work has been cited and discussed in the revised manuscript.

**Changes in the manuscript**

The approximation of neglecting the unsteadiness and the friction losses have been criticized in (33). A similar approach is undertaken here to calculate the pressure drop.

Why were these specific values of distensibility chosen? Fig 13 and 12 - the x axis is labeled differently, please use same notations.

**Authors’ Reply**

Experimentally, the afterload pump speeds were chosen in order to produce tube expansion within physiological range of aorta expansion during systole (120-160%). Experimental range was approximately 130 – 156%, which corresponded to distensibility range shown in Fig. 12. This has been added to the revised manuscript.

**Change in the manuscript**

The afterload pump speeds were chosen to produce tube expansion within physiological range of aorta expansion during systole (120-160%). It therefore resulted in achieving aorta expansion of 130-156% in the experiments.

Fig. 12 was edited to have the same *x* axis label as in Fig. 13. Also, the absolute value of the peak velocity ratio is now used in Fig. 12 in order to reflect the increasing influence of the negative centerline velocity with the rigid tube as compared to the decreasing influence of the negative centerline velocity with the compliant tube.

Fig 12: Plot representing the peak positive downstream centerline velocity, peak negative centerline velocity and absolute peak velocities ratio (peak negative to peak positive) as function of mock aorta distensibility for the compliant case where $\text{P}_{\text{ch}}\text{ = 103 mmHg}$.
