# Supplementary material for: Experimental investigation into the effect of compliance of a mock aorta on cardiac performance
Source: PLoS One. 2020 Oct 12;15(10):e0239604. doi: 10.1371/journal.pone.0239604 (PMC7549783; doi:10.1371/journal.pone.0239604)
Supplement: Attachment — Submitted filename: Authors_Rebuttal_PLOS1_review2_v3.docx [file pone.0239604.s002.docx]

# REPLY TO REVIEWER COMMENTS

Report on “Experimental investigation into the effect of compliance of a mock aorta on cardiac performance ".

Note: Reviewer’s comments have been copied from the email sent by the editor.

# Reply to comments from Reviewer #1

*Reviewer #1:*

Reviewer #1: The authors have responded to the comments and suggestions with sufficient detail and the paper is in publication-mode. However there are two minor points that need to be addressed:

**Authors’ Reply**

Thank you for reviewing the paper and the comments.

1. In the extended discussion regarding the cardiac performance, the added reference discusses a new metric (hydrodynamic efficiency) for cardiac performance and shows that it illustrates more details than ejection fraction; therefore, it is not a good reference for the importance of ejection fraction.

**Authors’ Reply**

The discussion was modified to well highlight the recent literature and its connection to the discussion.

**Change in the manuscript:**

While there is research in the literature that uses metrics such as hydrodynamic efficiency (22) to assess the physiological conditions of the heart, there is limited research into how the circulatory conditions affect heart performance in an ex-vivo setup.

2. In equations 2 and 3, density is missing in the acceleration terms.

**Authors’ Reply**

Density was added to the terms in equations 2 and 3.

# Reply to comments from Reviewer #2

*Reviewer #2:*

- New figure 5b that has been provided does not make sense. How come the velocity near the walls are much higher than the velocity near the center? During systolic phase (flow acceleration) the bulk flow should accelerate and then decelerate, with the maximum velocity nominally close to centerline. Which phase (time point) of systole is presented?

**Authors’ Reply**

Thank you for the time and specific comments.

An additional systole phase was added to provide further details on the flow dynamics downstream from the bi-leaflet aortic valve. The time stamp of the 3 phases is now shown on the velocity distribution as points (a), (b) and (c) in the figure below. For greater clarity, the velocity fields of the systole phases (a, b) are presented using the same velocity measurement scale whereas the diastole phase (c) has a different scale. It should be noted that the systole phase (a) corresponds to higher velocities on the lateral regions of the mechanical valve as compared to its central region. This is related to the flow behaviour of the 3 jets of the bi-leaflet valve which is also affected by the leaflets orientation (Pibarot and Dumesnil, 2009).

Pibarot P. and Dumesnil J.G. (2009) *“Prosthetic Heart Valves Selection of the Optimal Prosthesis and Long-Term Management”* Circulation, Volume 119, Issue 7, Pages 1034-1048

**Change in the manuscript:**

The three plots in Fig 5 are for the initial stage of the systole when the flow begins to accelerate, Fig 5(a), at the develop phase of the systole around the maximum velocity, Fig 5(b) and for the diastole where the average centerline velocity is approaching zero, Fig 5(c). The example vector data set in Fig 5(c) illustrates the presence of secondary flows that arise at the end of diastole when the flow is nearing the end of deceleration. It should be noted that the systole phase Fig 5(a) corresponds to higher velocities on the lateral regions of the mechanical valve as compared to its central region. This can be related to the flow behaviour of the 3 jets of the bi-leaflet valve which is also affected by the leaflets orientation as discussed in (64).

The points (a), (b) and (c) on Fig 10(a) are the time stamp of the 3 phases during the initializing of the systole, during the systole around the maximum centerline velocity and at the diastole for which the velocity vector fields are plotted in Fig 5(a).

**Fig 10 caption:** The points (a), (b) and (c) on Fig 10(a) are the time stamp of the 3 phases for which the velocity vector fields are plotted in Fig 5(a).

- Fig 10b - the pressure profile and flow rate profile are not physiological, when compared to expected aortic pressure. Please mark when systolic phase ends? How does flow rate not go down to zero at the end of systole? It slowly decays to zero. After systole, the flow rate has to be close to zero for the entirety of diastole, which is not being simulated here.

**Authors’ Reply**

According to the general representations of the three expected shapes of central aortic waveforms (type a, type b and type c), the waveform does have an expected physiological shape (but with higher than expected systolic peak pressures because of the LVAD).:

Our waveform shape as a type c waveform with a large and early arriving (during systole) reflected wave. The shape of our waveform is consistent with an expected physiological waveform, but the magnitudes are different as we have a higher systolic peak pressure. The position of systolic start and end are shown on Fig 6 in the manuscript.

With respect to flow, the plot represents the flow rate downstream of the aorta, and not the flow rate through the aorta. Downstream will see much steadier flow. We understand and agree with the reviewer that a flow waveform at the aortic root would look like the following plot. However, moving downstream (where we are taking the flow waveform), that systolic peak flow will be reduced and it won’t necessarily reach 0 during the systolic-diastolic transition. The high ejection pressures and velocities in our experiment, may contribute to slow decaying of downstream flow during diastole rather than reaching a high peak during systole then dropping to zero.

- And in addition, the end of normalized time (1.0) does not continue with start of normalized time (0), this is a major discrepancy in the presented data.

**Authors’ Reply**

The problem has occurred during the data processing. This has been corrected so the plots match the normalized pressure data. The actual pressure values were requested after the first review and were provided in Fig 10(b). Please note that the pressure data in Fig 10(b) is the absolute pressure and not the gauge pressure.

**Change in the manuscript:** a new plot for Fig 10(b) was generated as below.

- Fig 10a and 10b - do not provide the same information for both rigid and compliant case (normalized pressure or absolute pressure), providing a mix, does not allow for a fair comparison.

**Authors’ Reply**

Plots presented in Fig 10(a) and (b) are only for compliant tube. Fig 10(b) provided after the first review upon request of the respected reviewer as additional information for the actual pressure values and the flow rates. Therefore, it is not a mixed plot for rigid and compliant case.
